# UVA1 irradiation inhibits fibroblast proliferation and alleviates pathological changes of scleroderma in a mouse model

**DOI:** 10.1016/S1674-8301(12)60023-2

**Published:** 2012-03

**Authors:** Mei Ju, Kun Chen, Baozhu Chang, Heng Gu

**Affiliations:** Institute of Dermatology, Chinese Academy of Medical Science, Nanjing Jiangsu 210042, China.

**Keywords:** ultraviolet irradiation A1 (UVA1), scleroderma, mouse model, fibroblasts, proliferation

## Abstract

The purpose of the present study was to compare the effects of different doses of ultraviolet radiation A1 (UVA1) on human fibroblast proliferation and collagen level in a mouse model of scleroderma, so as to identify appropriate irradiation doses for clinical treatment of scleroderma. Monolayer from human fibroblasts was cultured *in vitro*, and a mouse model of scleroderma was established by subcutaneous injection of 100 µL of 400 µg/mL bleomycin into the back of BALB/c mice for 4 weeks. The mouse models and human fibroblasts were divided into UVA1-exposed (100, 60 and 20 J/cm^2^) and UVA-unexposed groups. At 0, 24 and 48 h after exposure, cell proliferation and levels of hydroxyproline and collagen were detected. UVA1 irradiation was performed 3 times weekly for 10 weeks, and the pathological changes of skin tissues, skin thickness and collagen level were observed after phototherapy. Cell proliferation and the levels of hydroxyproline and collagen were inhibited after phototherapy, and there was a significant difference between the UVA1-exposed cells and UVA1-unexposed cells (*P* < 0.001). In addition, UVA1 phototherapy improved dermal sclerosis and softened the skin, and there were significant differences between the high-dose UVA1 group and the model group, and the negative group (*P* < 0.05). It is concluded that UVA1 radiation can reduce cell proliferation, and decrease hydroxyproline and collagen levels in a dose-dependent manner *in vitro*. High-dose UVA1 phototherapy has marked therapeutic effect on scleroderma in the mouse model. Decreased collagen level may be related to the reduced number and activity of cells, as well as inhibition of collagen synthesis.

## INTRODUCTION

Scleroderma is a chronic connective tissue disorder of heterogeneous clinical manifestations often with a progressive course. The diffuse cutaneous form of the disease is characterized by thickening of the skin and visceral fibrosis, which may involve multiple internal organs like the lungs, gastrointestinal tract and heart. Sclerosis of the skin can lead to dysmorphism, contractures and restrictions of movement[1]. The mechanisms and pathogenesis of this complex disorder are not well understood to date, although the increased expression of collagen-producing fibroblasts in the skin has been demonstrated. Treatments with corticosteroids, immunomodulatory agents, ultraviolet (UV) radiation and vitamin D analogues are used to prevent the development of new skin and organs in the early stage and improve the symptoms in the advanced stage.

UVA1 (wavelength: 340-400 nm) phototherapy is a new physical therapy in recent years, which is effective in the treatment of scleroderma and other connective tissue diseases[2],[3]. UVA1, apart from its well-known effects on collagen metabolism, is thought to be related to the regulation of immune function and infiltration of T-cells in the dermis by induction of various cytokines and soluble factors[4],[5]. However, its detailed mechanism of action has remained unknown till now, and the optimal dose of UVA1 irradiation still has not been determined. Stege and colleagues[6] found that high-dose (130 J/cm^2^) UVA1 irradiation was superior to low-dose (20 J/cm^2^) UVA1 therapy in the treatment of localized scleroderma, and the effectiveness was dose dependent. However, others[3],[7]-[9] reported that medium-dose (60 J/cm^2^) and low-dose (20-30 J/cm^2^) UVA1 therapy was similarly effective against localized scleroderma, and there was no significant difference in clinical scores between the medium-dose (60 J/cm^2^) and low-dose (20 J/cm^2^). However, medium-dose UVA1 phototherapy provided better long-term results than low-dose UVA1 phototherapy in localized scleroderma as shown by ultrasound assessment[10]. The above arguments may be related to lack of a control group and the small sample size. Scleroderma clinical trials are difficult to carry out due to long disease duration, severe conditions, thorny issues in the treatment of the disease and poor compliance. The purpose of this study was to investigate the mechanism of UVA1 phototherapy and the appropriate dose of irradiation for clinical treatment of scleroderma in a mouse model[11].

## MATERIALS AND METHODS

### Reagents and apparatus

Bleomycin (Nippon Kayaku Co., Ltd, Japan); the 3-(4,5-dimethylthiazol-2-yl)-5(3-carboxymethoxyphenyl)-2(4-sulfophenyl)-2H-terazolium (MTT) assays (Shanghai Maisha Biotechnology Co., Ltd, Shanghai, China); dimethyl sulfoxide (DMSO, Nanjing Chemical Reagent Co., Ltd, Nanjing, China); trypsin (AMRERSCO, Solon, OH, USA); RPMI 1640 medium (GIBCO/BRL, Gaithersburg, MD, USA); fetal bovine serum (Hangzhou Sijiqing Biological Engineering Materials Co., Ltd, Hangzhou, China); hydroxyproline assay kit (Nanjing Jiancheng Bioengineering Institute, Nanjing, China).

Philips TL/10 UVA Phototherapy lamp (output wavelength, 340-400 nm; effective exposure area, 220 mm×325 mm; irradiation time, 1 s to 60 min; exposure intensity, > 30 mW/cm^2^; Sigma-Aldrich, (Shanghai, China); UVA radiation meter (Sigma-Aldrich); DG3022A enzyme-linked immunosorbent assay (ELISA) reader (Nanjing Huadong Electronics Group Co., Ltd, Nanjing, China); BN4-311CO_2_ incubator (Espec, Osaka, Japan); inverted microscope (Olympus, Tokyo, Japan); PK-8B type electric water heater (Shanghai Jinghong Laboratory Instrument Co., Ltd, Shanghai, China); 7550 UV-Vis spectrophotometer (Shanghai Analytical Instrument Factory, Shanghai, China).

### Animals and groups

The study protocol was approved by the institutional review board at the authors' affiliated institution and animal study was carried out in accordance with the established institutional guidelines regarding animal care and use. Sixty 6-week-old female mice of the BALB/c strain, each weighing 18-22 g, were purchased from Yangzhou University [Animal production license number: SCXK (Su) 2002-0009], and given free access to food and water. Mice were randomly assigned to 6 groups: 1) the mice in the control group were normal mice without any treatment and sacrificed by cervical dislocation after 4 weeks; 2) the model group, in which mice received a daily hypodermical injection of 100 µL BLM (400 µg/mL) into the back for 4 weeks to establish the model of scleroderma, which were sacrificed by cervical dislocation at d 29; 3) higher UVA1 dose group, in which the animals with scleroderma were treated with high dose UVA1 (100 J/cm^2^); 4) the medium UVA1 dose group, in which the animals with scleroderma were treated with 60 J/cm^2 UVA1^; 5) the low UVA1 dose group, in which the animals with scleroderma were treated with 20 J/cm^2^ UVA1; 6) the negative control group were animals with scleroderma without any treatment and sacrificed by cervical dislocation after 10 weeks. Phototherapy was performed 3 times weekly for 10 weeks, and then the mice were sacrificed by cervical dislocation for group 3,4, and 5.

### Primary fibroblast culture and subculture

Human fibroblasts from the foreskin of healthy adolescent males who underwent circumcision were cultured by primary dispersal cell culture method. The fibroblasts were digested with 0.25% trypsin when the cells became confluent, which were then centrifuged at 800 *g* for 10 min. Cells at a concentration of 1×10^6^ cells/mL were seeded into 30 cm^2^ culture flasks and supplemented with 4 mL of culture medium, which were then cultured at 37°C in an incubator containing 5% CO_2_. The third to fourth passage cells were used in this experiment.

### Monolayer cell culture

Cell suspension was prepared when the third to fourth passage fibroblasts were grown to 90% confluence, and then 0.1 mL of cell suspension at a density of 3×10^5^ cells/mL was seeded into 96- or 24-well plates, which was then cultured at 37°C in an incubator containing 5% CO_2_.

### Establishment of mouse model of scleroderma

Experimental mouse models of scleroderma were established as described below. Briefly, 100 µL of 400 µg/mL bleomycin was injected subcutaneously in the dorsal skin of BALB/c mice for 4 weeks. Following injection, histological examination and the concentrations of hydroxyproline and collagen were detected to validate the success of the models.

### UVA1 phototherapy

Mice were exposed to high, medium or low dose UVA1 phototherapy 3 times weekly for 10 weeks, respectively. Fibroblasts were divided into three UVA1-exposed groups and the control group, and the irradiation dose was the same as that used in the mouse models.

### MTT assays

At 0, 24 and 48 h after phototherapy, 20 µL MTT solution was added into each well and cultured for another 4 h. Then, each well was added with 150 µL DMSO and vortexed for 10 min to dissolve the crystals. The absorbance (A value) of each well was determined at 490 nm.

### Determination of hydroxyproline and collagen levels

At 0, 24 and 48 h after phototherapy, fibroblasts were digested with 0.25% trypsin and were rendered into single cell suspension at a concentration of 1×10^5^ cells/mL. The cell density of the suspension was counted under a microscope, and hydroxyproline level in 250 µL cell suspension was determined with the hydroxyproline assay kit. Collagen concentration was calculated using the following formula: collagen concentration (µg/mL) = Sample hydroxyproline concentration ×7.46.

### Histological observation

Mice were sacrificed by cervical dislocation after the experiment. Two pieces of skin were taken from the injection sites, and then stained with hematoxylin and eosin (H&E). The pathological changes of skin tissues and thickness of skin (epidermis + dermis) were observed. The contents of hydroxyproline and collagen were detected the same as fibroblasts from another piece of skin.

### Statistical analysis

All data were expressed as mean±standard deviation (SD), and all statistical analyses were performed using the statistical software Statistical Package for the SPSS 11.0 (SPSS Inc., Chicago, IL, USA). Differences were tested for statistical significance with two-way analysis of variance (ANOVA), and Student-Newman-Keuls (SNK) method was used for multiple comparisons. A *P*-value < 0.05 was considered significant.

## RESULTS

### *In vitro* proliferation of fibroblasts after UVA1 irradiation

Fibroblast growth was inhibited following UVA1 irradiation and the inhibition gradually increased in a dose-dependent manner, with the most critical damage at 24 h in the high-dose UVA1 group. Statistical difference was observed at different times (*F* = 49.672, *P* < 0.001) and different doses (*F* = 25.580, *P* < 0.001, ***Table 1***).

**Table 1:** OD value of fibroblasts after UVA1 irradiation

Group	0 h	24 h	48 h
Control	0.19 ± 0.01	0.16 ± 0.01	0.30 ± 0.01
Low-dose UVA1	0.20 ± 0.00	0.05 ± 0.02*	0.16 ± 0.03*
Medium-dose UVA1	0.11 ± 0.03*	0.06 ± 0.03*	0.17 ± 0.03*
High-dose UVA1	0.05 ± 0.03*	0.03 ± 0.02*	0.19 ± 0.02*

*Compared to the control group, *P* < 0.01.

### Morphological changes of fibroblasts after UVA1 irradiation

**Fig. 1:**
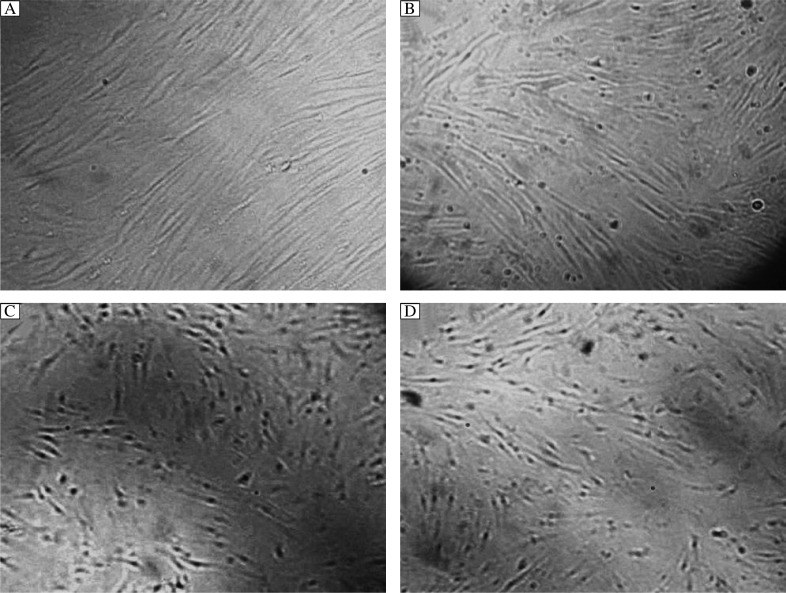
The morphology of fibroblasts between normal and UVA1-exposed cells(×100). Morphology of normal fibroblasts (A) fibroblasts at 24 h after low (B), medium (C) and high (D) dose of UVA1 irrdation. The fibroblasts after UVA1 irradiation appeared swollen, broken and round, and the damage of high dose UVA1 was most critical.

Under an inverted microscope, normal fibroblasts appeared like rod or spindle-shaped, and interconnected into spiral or turbine-shaped structure, with clear and plump bodies (***Fig. 1A***). Cells exposed to 100, 60 and 20 J/cm^2^ of UVA1 were damaged to different degrees, amd they appeared swollen, broken and round. Some cells lost connection with adjacent cells, and the intercellular gap was widened. The most critical damage was observed at 24 h after high-dose UVA1 irradiation (***Figs. 1C*** to ***1D***). At 48 h after irradiation, cells in the low-dose UVA1 group almost returned to the normal spindle-like structure. There were different degrees of damages in cells of the medium-dose and high-dose UVA1 groups, but the damage was slightly improved.

### Hydroxyproline and collagen levels of fibro-blasts after UVA1 irradiation

Compared with those of the control group, the levels of hydroxyproline and collagen decreased after UVA1 phototherapy, and statistical differences were observed between the UVA1-exposed and UVA1-unexposed cells (*P* < 0.001). Moreover, UVA1 phototherapy decreased hydroxyproline and collagen levels in a dose-dependent manner, and there were statistical differences at various doses of UVA1 (***Table 2***).

**Table 2:** Hydroxyproline and collagen levels in fibroblasts after UVA1 irradiation

Group	Hydroxyproline (µg/mg)			Collagen (µg/mL)		
	0 h	24 h	48 h	0 h	24 h	48 h
Control	6.61 ± 0.19	7.85 ± 0.07	13.25 ± 0.62	49.09 ± 1.14	58.55 ± 0.53	98.84 ± 4.66
Low-dose UVA1	6.04 ± 0.09	4.67 ± 0.07	9.95 ± 0.20	45.04 ± 0.71	34.85 ± 0.49	74.25 ± 1.49
Medium-dose UVA1	5.41 ± 0.22	4.27 ± 0.05	8.34 ± 0.21	40.10 ± 1.23	31.88 ± 0.35	62.19 ± 1.57
High-dose UVA1	4.83 ± 0.06	3.78 ± 0.11	7.11 ± 0.54	36.02 ± 0.43	27.17 ± 2.43	53.06 ± 4.03
*F* (time)		107.739			108.268	
*P* (time)		*P* < 0.001			*P* < 0.001	
*F* (dose)		40.087			40.294	
*P* (dose)		*P* < 0.001			*P* < 0.001	

### Pathological changes in the skin of mice after phototherapy

**Fig. 2:**
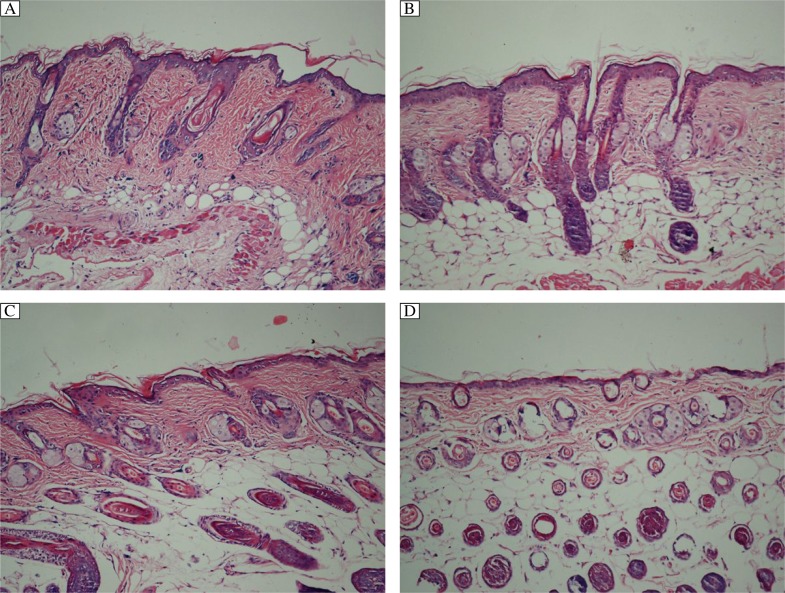
H&E staining of skin of mice in the model group (A) and mice irradiated 30 times with low (B), medium (C) and high (D) dose UVA1 (×100). The thickness of dermis gradually thinned after UVA1 irradiation, especially high dose UVA1 group, and no significant proliferation and hair follicles were also seen in the fat layer structure.

H&E staining showed that dermal sclerosis was characterized by thickened derma and homogenous collagen bundles in the model group (***Fig. 2A***), and thin skin and fewer collagen fibers were present in the control group. After phototherapy, the histopathological examination of skin from the UVA1-irradiated mice revealed thinning of the dermis. No significant proliferation of collagen fibers and hair follicles were seen in the fat layer structure, and the most obvious changes were found in the high-dose UVA1 group (***Figs. 2B*** to ***2D***). However, increased dermal thickness and fiber density were observed in the control group after 10 weeks, which was similar to the model group.

### Changes in dermal thickness and collagen level after phototherapy

UVA1 phototherapy exhibited significant therapeutic effect in the mouse models, which could improve dermal sclerosis and soften skin. The dermis became thinner as hydroxyproline and collagen levels were gradually reduced. There were significant differences in dermal thickness, hydroxyproline and collagen levels between the high-dose UVA1 and the model and negative control groups (*P* < 0.05). In the 3 groups receiving UVA1 phototherapy, high-dose UVA1 phototherapy was more effective than the medium-dose and low-dose treatment (*P* < 0.01, ***Table 3***).

**Table 3:** Hydroxyproline and collagen levels of mice after UVA1 irradiation

Group	*n*	Dermal thickness (µm)	Hydroxyproline (µg/mg)	Collagen (µg/mg)
Control group	10	6.96 ± 0.64	3,442.50 ± 632.10	25,681.05 ± 4,715.53
Model group	10	10.68 ± 2.29**	5,041.10 ± 1720.92**	37,606.61 ± 12,838.04**
High-dose UVA1	10	6.82 ± 0.68	2,692.80 ± 978.09	20,088.29 ± 7.296.59
Medium-dose UVA1	10	9.30 ± 1.02**	4,279.50 ± 672.93**	31,925.07 ± 5,020.05**
Low-dose UVA1	10	10.28 ± 2.22**	3,989.70 ± 1407.43*	29,763.16 ± 10,499.448*
Negative control group	10	9.37 ± 1.60**	4,735.80 ± 1351.71**	35,329.07 ± 1,003.74**

*Compared with high-dose UVA1 group, *P* < 0.05; **Compared with high-dose group, *P* < 0.01.

## DISCUSSION

Scleroderma is a chronic disease of unknown cause, characterized by diffuse fibrosis; degenerative changes; vascular abnormalities in the skin (scleroderma), articular structures, and internal organs, especially the esophagus, intestinal tract, lung, heart, and kidney. Several studies have demonstrated that excessive collagen deposition was due to fibroblast dysfunction in scleroderma. UVA1 (wave length between 340 and 400 nm) phototherapy is a novel physical therapy used in recent years[12], but its detailed mechanism so far remains unknown. It is thought that UVA1 suppresses fibroblast proliferation, inhibits collagen synthesis and promotes collagen degradation, induces T cell apoptosis and regulates the expression of transcription growth factor (TGF)-β, interferon-γ (IFN-γ), matrix metalloproteases (MMP), interleukin-6 (IL-6), and IL-8[13]-[16]. However, the interaction and roles of these factors are uncertain.

Recent studies found that UVA1-irradiated fibroblasts exhibited abnormal morphology and ceased growth. Moreover, cell damage was positively correlated with the radiation dose. UVA1 induces apoptosis of fibroblasts by reactive oxygen species (ROS), in which singlet oxygen is the most important member[17]-[20]. It has been demonstrated that low-dose (20 J/cm^2^) UVA1 irradiation could inhibit fibroblast proliferation in a dose-dependent manner[21]. Similarly, our findings showed that high-dose (100 J/cm^2^) UVA1-irradiated fibroblasts appeared immediately edemous, broken, and round. The most obvious damages were observed at 24 h after irradiation with high-dose UVA1 and recovered gradually at 48 h after irradiation. However, cells could not restore the normal structure.

In order to compare the effects of different doses of UVA1 irradiation on fibroblasts *in vitro*, MTT assay was performed to detect the proliferation of fibroblasts exposed to different doses of UVA1 at different times. The results showed that non-irradiated fibroblasts were always in proliferation, and the proliferation index at 24 h was slightly lower than that at 0 h, probably due to PBS treatment. A single dose of UVA1 irradiation inhibited fibroblast growth, obviously at 24 h after irradiation. Cells were gradually recovered at 48 h after irradiation. There were statistically significant differences between different doses of UVA1 groups. The current study indicated that UVA1 irradiation inhibited human skin fibroblast proliferation in a dose-dependent manner.

In this study, we also observed changes of hydroxyproline and collagen levels in fibroblasts at different time points after irradiation with different doses of UVA1. The results showed that there were significant differences in the inhibition of hydroxyproline and collagen levels in fibroblasts irradiated with single dose of UVA1 compared with the UVA1-unexposed cells. The strongest inhibition was found at 24 h and gradually recovered at 48 h after phototherapy. Moreover, the inhibition was dose-dependent. Significant difference was detected in various doses of UVA1 radiation groups, and there were also significant differences in all UVA1 irradiation groups compared with the control groups (*P* < 0.001). Lei *et al*.[22] investigated fibroblast proliferation by the MTT method, and collagen biosynthesis by ^3^H-proline incorporation after UVA light irradiation for 2 d. The results showed that collagen biosynthesis in UVA-exposed cells was decreased compared with that of the unexposed cells (*P* < 0.001), and the decrease was not caused by the reduced number of fibroblasts, but because of the reduced capacity of collagen biosynthesis. Our findings showed that that the reduced collagen level after UVA1 irradiation could be related to the decreased number of cells and activity, as well as inhibition of collagen synthesis, which was consistent with other studies[23].

In recent years, *in vitro* study indicated that UVA1 irradiation could up-regulate the expression of MMP-1, MMP-2 and MMP-3 mRNA in fibroblasts, and UVA1 irradiation up-regulated MMP-1 mRNA expression in a dose-dependent manner. MMP-1 has been shown to play an important role in collagen degradation, and the mechanism of UVA1 irradiation was dependent on different subtypes of MMP[24],[25]. Yin and colleagues[26] compared MMP-1 expression after UVA1 phototherapy in normal fibroblasts and fibroblasts with systemic scleroderma, and found increased expression in both groups, but higher expression was observed in fibroblasts with systemic scleroderma than in normal fibroblasts. Another *in vivo* study revealed that MMP-1 expression in dermal fibroblasts was markedly increased, and the levels of type I and type III collagen were significantly decreased in the lesions of scleroderma patients after high-dose UVA1 irradiation[27]. These *in vivo* and *in vitro* studies showed that the mechanism of UVA1 phototherapy could be related to promoting MMP-1 expression, reducing collagen degradation and inhibiting collagen synthesis.

In the present study, we also established mouse models of scleroderma as previously described[28],[29], and observed the difference of skin thickness. The levels of hydroxyproline and collagen were maintained for 10 weeks post injection without any intervention in the control and mouse model groups, which was longer than previously reported[28],[30]. It has been shown that the models induced by bleomycin were successful and reproducible. The present study found that high-dose UVA1 phototherapy was more effective than medium-dose and low-dose UVA1. Although medium-dose and low-dose UVA1 could also improve skin thickness, there was no significant difference compared with the model group, which was the same as that Stege's study[6].

Low-dose UVA1 phototherapy could damage cells in vitro; however, only high-dose UVA1 phototherapy had significant therapeutic effect in mouse models. It is indicated that *in vitro* results were not consistent with those of *in vivo* studies, especially in the human body, which were more complex. Moreover, the adverse effects of UVA1 phototherapy, including short-term adverse effects like itching, erythema, burning sensation and pain and long-term adverse effects like photoaging and skin cancer were not observed, either. Therefore, further studies to explore the mechanisms and therapeutic effects of UVA1 phototherapy in scleroderma patients seem justified.
